# Formation of the *Legionella* Replicative Compartment at the Crossroads of Retrograde Trafficking

**DOI:** 10.3389/fcimb.2017.00482

**Published:** 2017-11-24

**Authors:** Kevin Bärlocher, Amanda Welin, Hubert Hilbi

**Affiliations:** Institute of Medical Microbiology, University of Zürich, Zurich, Switzerland

**Keywords:** *Dictyostelium discoideum*, effector protein, host-pathogen interaction, pathogen vacuole, retrograde transport, retromer, sorting nexin, type IV secretion

## Abstract

Retrograde trafficking from the endosomal system through the Golgi apparatus back to the endoplasmic reticulum is an essential pathway in eukaryotic cells, serving to maintain organelle identity and to recycle empty cargo receptors delivered by the secretory pathway. Intracellular replication of several bacterial pathogens, including *Legionella pneumophila*, is restricted by the retrograde trafficking pathway. *L. pneumophila* employs the Icm/Dot type IV secretion system (T4SS) to form the replication-permissive *Legionella*-containing vacuole (LCV), which is decorated with multiple components of the retrograde trafficking machinery as well as retrograde cargo receptors. The *L. pneumophila* effector protein RidL is secreted by the T4SS and interferes with retrograde trafficking. Here, we review recent evidence that the LCV interacts with the retrograde trafficking pathway, discuss the possible sites of action and function of RidL in the retrograde route, and put forth the hypothesis that the LCV is an acceptor compartment of retrograde transport vesicles.

## Components of the retrograde trafficking pathway

Eukaryotic cells employ complex endomembrane systems to process internalized material and transport newly synthesized proteins from the endoplasmic reticulum (ER) via the Golgi apparatus to target organelles and membranes (Johannes and Wunder, 2011). The sub-compartments of these endocytic and secretory (anterograde) pathways, respectively, communicate through vesicular trafficking. Endosomes represent sorting hubs, where the endocytic, secretory and lysosomal pathways meet and diverge.

**Figure 1:**
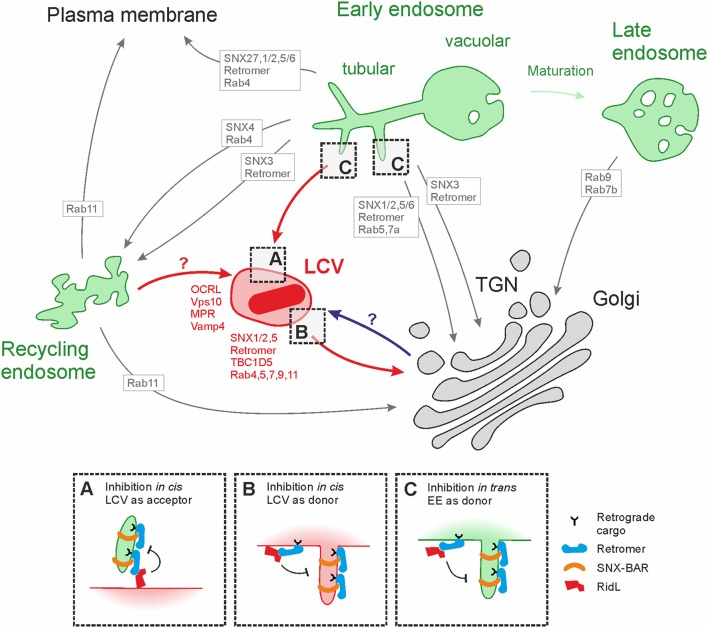
Positioning of the *Legionella*-containing vacuole in the retrograde route. Tubular/vacuolar early endosomes (EE; green) represent a sorting hub, where endosomal, recycling and retrograde trafficking pathways emerge to deliver cargo to late and recycling endosomes, the plasma membrane or the *trans*-Golgi network (TGN). The pathways employ distinct transport machineries (indicated with gray arrows and boxes), including small Rab GTPases, the PI 5-phosphatase OCRL, the retromer and sorting nexins (SNXs). Some of these retrograde trafficking components as well as retrograde cargos (MPR, sortilin) are also found on the LCV (indicated in red), suggesting that the pathogen vacuole is participating in these pathways. Possible retrograde transport routes involving the LCV are shown with red arrows. Inhibition of retrograde trafficking by RidL targeting the retromer might occur *in cis*, considering the LCV as (A) acceptor or (B) donor compartment for retrograde transport vesicles, or (C) *in trans* with the endosome as donor compartment. A possible role in LCV formation of late secretory (anterograde) trafficking emanating from the TGN is shown in blue. For details see text.

Retrograde transport from endosomes back to the ER maintains organelle integrity, prevents lysosomal degradation and recycles cargo receptors of the secretory transport machinery (Johannes and Popoff, 2008; Seaman, 2012; Burd and Cullen, 2014; Lu and Hong, 2014; Progida and Bakke, 2016). Dependent on the cargo and various endogenous factors, retrograde transport can occur from different endosomal donor compartments such as early, late and recycling endosomes (EE, LE, RE) as well as the tubular endosomal network (TEN) (Bonifacino and Rojas, 2006; Figure 1). Each membrane is defined by the presence of specific mono- or polyphosphorylated phosphoinositide (PI) lipids and small GTPases of the Rab family, which together determine the spatio-temporal recruitment of specific transport machineries (Di Paolo and De Camilli, 2006; Wandinger-Ness and Zerial, 2014).

The PI 5-phosphatase OCRL (Oculocerebrorenal syndrome of Lowe) is required for retrograde trafficking from EEs to the *trans*-Golgi network (TGN) and for receptor recycling between endosomes and the plasma membrane (PM) (Noakes et al., 2011; Vicinanza et al., 2011; Sharma et al., 2015). OCRL and its *Dictyostelium* homolog Dd5P4 (*D. discoideum* 5-phosphatase 4) hydrolyze PtdIns(4,5)*P*_2_ and PtdIns(3,4,5)*P*_3_ to yield PtdIns(4)*P* and PtdIns(3,4)*P*_2_ (Zhang et al., 1995; Loovers et al., 2007). OCRL binds to clathrin-coated pits (Ungewickell et al., 2004; Mao et al., 2009) as well as to a range of Rab GTPases including Rab5 on EEs (Hyvola et al., 2006; Fukuda et al., 2008; Hou et al., 2011).

Cargo shuttled via the retrograde endosome-to-TGN pathway comprises many different membrane and soluble proteins as well as lipids. Membrane protein cargos include Vps10 domain family cargo receptors such as cation-dependent or cation-independent mannose 6-phosphate receptor (CD- or CI-MPR) and sortilin/Vps10 (Seaman et al., 1997; Seaman, 2007). Soluble protein cargos include well-studied bacterial AB toxins, such as Shiga toxin (STxB) and cholera toxin (CTxB), which bind to the cell surface glycosphingolipids Gb3 (Lingwood, 1993) or GM1 (Kuziemko et al., 1996), respectively, and hijack the retrograde trafficking pathway to reach the host cell cytosol.

A key component of retrograde trafficking is the retromer complex consisting of a Vps26-Vps29-Vps35 heterotrimer (herein referred to as “retromer”) and different combinations of sorting nexin (SNX) proteins, which harbor a PI-binding PX (phox homology) domain (Cullen and Korswagen, 2011; Teasdale and Collins, 2012). Additional factors required for retromer recruitment are retrograde cargo receptors and the small GTPase Rab7a, a regulator of late endosome dynamics, in its activated, GTP-bound form (Rojas et al., 2008; Lucas et al., 2016). The binding of Rab7a to the membrane is negatively regulated by the GTPase activating protein (GAP) TBC1D5, which itself binds to Vps29 (Seaman et al., 2009; Harrison et al., 2014; Jia et al., 2016).

The retromer interacts with the membrane-associated heterodimer of SNX-BAR subfamily members SNX1/2 and SNX5/6, which harbor an additional dimerization and membrane curvature sensing BAR (Bin/Amphiphysin/Rvs) domain (Rojas et al., 2007; Wassmer et al., 2007; Frost et al., 2009). Together with further accessory proteins including the WASH complex (Gomez and Billadeau, 2009; Harbour et al., 2010), EHD1/dynamin large GTPases (Gokool et al., 2007) and p150^glued^/microtubule motor dynein (Wassmer et al., 2009), the retromer-SNX-BAR complex gives rise to tubule formation, elongation, scission, and transport of vesicles along microtubules to receiver compartments (Carlton et al., 2004; van Weering et al., 2012).

SNXs also include the SNX-PX subfamily member SNX3, which comprises of only a PX domain, binds to Vps35-Vps26 and is necessary for retromer recruitment to the endosomal membrane and the recognition of some cargo (Chen et al., 2013; Harrison et al., 2014; Lucas et al., 2016). The SNX-FERM subfamily member SNX27 interacts with cargo, Vps26, SNX1-SNX2, and the WASH complex (Temkin et al., 2011; Steinberg et al., 2013; Gallon et al., 2014). The SNX27-retromer complex is necessary for Rab4-dependent endosome-to-PM recycling of different cargos including many transporters such as glucose transporter 1 (GLUT1) (Steinberg et al., 2013).

After un-coating of the transport carrier, different components such as tethering factors and SNARE (soluble *N*-ethylmaleimide-sensitive factor attachment receptor) proteins are necessary for selective delivery to target membranes (Progida and Bakke, 2016). Thus, eukaryotic cells employ many distinct types of cargo-specific retrograde trafficking machinery to retrieve proteins and lipids from endosomes for recycling and to maintain organelle integrity.

## Formation and maturation of the *Legionella*-containing vacuole

The opportunistic pathogen *L. pneumophila* is the clinically most relevant *Legionella* species causing the severe pneumonia Legionnaires' disease (Bangsborg, 1997; Newton et al., 2010). *Legionella* spp. are ubiquitous Gram-negative environmental bacteria that colonize both natural and technical water systems, periodically causing disease outbreaks tracing back to cooling towers, whirlpools and showers. In the environment, *L. pneumophila* survives in both extra- and intracellular niches, i.e., the bacteria colonize biofilms, but also parasitize free-living protozoa (Hilbi et al., 2011). Natural hosts for *L. pneumophila* include *Acanthamoeba, Hartmannella*, and *Tetrahymena* species, and the bacterium also thrives within *Dictyostelium discoideum*, a commonly employed protozoan experimental host (Hoffmann et al., 2014b).

Through an evolutionarily conserved mechanism, *L. pneumophila* avoids killing by protozoan and mammalian phagocytes alike, allowing replication within a distinct intracellular compartment termed the *Legionella*-containing vacuole (LCV) (Isberg et al., 2009; Hubber and Roy, 2010; Hilbi and Haas, 2012; Sherwood and Roy, 2013; Finsel and Hilbi, 2015). Accidental infection of humans occurs through the inhalation of contaminated aerosols, although recently, a probable person-to-person transmission was reported for the first time (Correia et al., 2016). If the alveolar macrophages fail to eliminate the invading bacteria through cell-autonomous processes, intracellular replication of the bacteria will ensue, possibly culminating in a severe pneumonia (Simon and Hilbi, 2015).

The formation of the LCV is a complex process governed by the Icm/Dot type IV secretion system (T4SS). This T4SS injects more than 300 different “effector” proteins into the host cell, dictating every step from uptake to interference with various vesicle trafficking pathways and autophagy, interaction with the ER, and finally escape from the host cell (Finsel and Hilbi, 2015; Ensminger, 2016). To this end, the Icm/Dot-translocated proteins subvert numerous host cell targets, including PI lipids, which are used by several effectors as membrane anchors, and small GTPases (Haneburger and Hilbi, 2013; Rothmeier et al., 2013; Finsel and Hilbi, 2015). LCVs avoid luminal acidification and fusion with lysosomes, but continuously and extensively communicate with multiple vesicle trafficking routes (Horwitz, 1983; Urwyler et al., 2009a; Xu et al., 2010; Zhao et al., 2017).

Interaction with the endocytic pathway is indicated by the presence of the small GTPases Rab5a, Rab7a, and Rab21 on the LCV membrane (Urwyler et al., 2009b; Hoffmann et al., 2014a). Moreover, LCVs acquire PtdIns(3)*P* within 1 min of bacterial uptake, and gradually lose this PI lipid within 2 h (Weber et al., 2014). In the endocytic pathway, PtdIns(3)*P* is a crucial regulator, necessary for recruitment of early endosomal antigen 1 (EEA1) and for downstream events leading to fusion of the phagosome with bactericidal lysosomes (Stenmark, 2009).

A hallmark of LCV maturation is the intimate interaction of the pathogen vacuole with the ER (Swanson and Isberg, 1995; Lu and Clarke, 2005; Robinson and Roy, 2006), as a consequence of Rab1-dependent recruitment of secretory vesicles at ER exit sites (Kagan and Roy, 2002; Arasaki and Roy, 2010; Arasaki et al., 2012). PtdIns(4)*P* is a major regulator of secretory vesicle trafficking through the Golgi apparatus (Jean and Kiger, 2012), required for late steps of endocytosis (Jeschke et al., 2015) and present on LCVs (Weber et al., 2006, 2014). In fact, PtdIns(4)*P* transiently localizes to LCVs independently of the Icm/Dot T4SS immediately following bacterial uptake, but is then rapidly cleared. Over the following 2 h, PtdIns(4)*P* again accumulates on LCVs in an Icm/Dot-dependent manner, preceding attachment of the ER (Weber et al., 2014). In addition to Rab1, several other small GTPases involved in the secretory pathway are present on the LCV (Urwyler et al., 2009b; Hoffmann et al., 2014a). Of these, Rab8a, Rab10, and Rab32, all implicated in Golgi to endosome trafficking, promote intracellular replication of *L. pneumophila* (Hoffmann et al., 2014a). Thus, *L. pneumophila* exploits the secretory trafficking pathway to promote formation of the replication-permissive LCV.

## Restriction of intracellular replication of *L. pneumophila* by retrograde trafficking

A number of intracellular bacterial pathogens subvert retrograde trafficking (Personnic et al., 2016). These include the obligate intracellular pathogen *Chlamydia trachomatis* (Aeberhard et al., 2015; Mirrashidi et al., 2015), facultative intracellular *Salmonella enterica* serovar Typhimurium (McGourty et al., 2012), and *Coxiella burnetii* (McDonough et al., 2013).

An early study using *D. discoideum* already implicated a role for retrograde trafficking during intracellular replication of *L. pneumophila* (Weber et al., 2009). This study revealed that the PI 5-phophatase OCRL/Dd5P4 restricts intracellular growth of *L. pneumophila*, while promoting the accumulation of ER on LCVs as well as the transition from “tight” to “spacious” pathogen vacuoles (Weber et al., 2009). In a more recent study, depletion of individual components of the retrograde machinery, such as the retromer subunits Vps26a/Vps26b or Vps29, or the retrograde cargo CI-MPR, led to increased intracellular replication of *L. pneumophila* (Finsel et al., 2013). Among the small Rab GTPases implicated in retrograde trafficking, Rab5a but not Rab7a, Rab9a or Rab11a restrict intracellular replication (Hoffmann et al., 2014a). Furthermore, in *D. discoideum* the TBC1D5 homolog promotes intracellular growth of *L. pneumophila* (Bärlocher et al., 2017). Since mammalian TBC1D5 functions as a Rab7 GAP and negatively regulates Rab7a, which is implicated in retrograde trafficking, these findings corroborate a function of the retrograde pathway in restricting *L. pneumophila* replication.

## Interaction of LCVs with the retrograde trafficking pathway

Proteomics analysis of isolated LCVs from infected protozoan and mammalian host cells revealed components of the retrograde trafficking pathway including small GTPases (Shevchuk et al., 2009; Urwyler et al., 2009b; Hoffmann et al., 2014a; Herweg et al., 2015; Schmölders et al., 2017). Whereas the late endosomal marker Rab9 is present on *L. pneumophila* wild-type as well as Δ*icmT* mutant-containing vacuoles, Rab4, Rab5, Rab7, and Rab11 are recruited to LCVs in an Icm/Dot-dependent manner (Finsel et al., 2013; Hoffmann et al., 2014a; Bärlocher et al., 2017). Rab4 and Rab11 associate predominantly with EE/RE or with RE/TGN, and are involved in EE/RE-to-PM or RE-to-PM/TGN trafficking, respectively (Zerial and McBride, 2001; Miserey-Lenkei et al., 2007; Figure 1).

With respect to retromer components, Vps26, Vps29, and Vps35 as well as SNX1, SNX2, SNX5, and TBC1D5 can be detected on LCVs (Finsel et al., 2013; Bärlocher et al., 2017). Moreover, retrograde cargos including Vps10, MPR, and Vamp4 are present on the pathogen vacuole (Finsel et al., 2013; Hoffmann et al., 2014a; Figure 1). The CI-MPR is shuttled between endosomes and the TGN in retromer-, SNX1/2- as well as Rab7b- and Rab9-dependent pathways (Lombardi et al., 1993; Arighi et al., 2004; Rojas et al., 2007; Progida et al., 2010, 2012). The SNARE Vamp4 is a retrograde cargo predominantly found at the TGN, which cycles to the cell surface and back via EE/RE (Tran et al., 2007).

The *L. pneumophila* Icm/Dot substrate RidL (Retromer interactor decorating LCVs) was identified as a binding partner of the retromer complex, and the effector protein promotes intracellular bacterial replication (Finsel et al., 2013). Upon infection of *D. discoideum* or macrophages, RidL decorates the LCV membrane and accumulates preferentially at the bacterial poles. Pulldown and protein-lipid overlay experiments revealed that RidL interacts specifically with the retromer Vps29 subunit and the PI lipid PtdIns(3)*P*, respectively. Ectopically produced RidL inhibits retrograde trafficking of fluorescently labeled STxB in HeLa cells, as evidenced by reduced Golgi localization of the toxin. Furthermore, in macrophages infected with wild-type *L. pneumophila*, retrograde trafficking of fluorescently labeled CTxB is impaired in a RidL-dependent manner. In presence of RidL, CTxB colocalizes with an EE/RE marker (transferrin receptor), but not with markers for lysosomes (dextran) or the Golgi apparatus (GM130). In absence of RidL, however, the toxin colocalizes with both EE/RE and Golgi markers, but not with the lysosome marker, consistent with a RidL-dependent inhibition of retrograde trafficking at endosome exit sites (Finsel et al., 2013).

A recent study revealed that the 29 kDa N-terminal domain of RidL (RidL_2*-*281_) adopts a new “foot-like” fold comprising a protruding hydrophobic β-hairpin at its “heel,” which interacts with Vps29 (Bärlocher et al., 2017). In HeLa cells, the fragment RidL_9*-*258_ co-localizes with the retromer complex and displaces the Rab7 GAP TBC1D5 from the retromer. Similarly, RidL translocated by *L. pneumophila* reduces the amount of TBC1D5 on LCVs during infection of *D. discoideum*. Thus, displacement of TBC1D5 from the Vps29 retromer subunit by the hydrophobic β-hairpin of RidL might contribute to effector function.

## The LCV as an acceptor compartment in the retrograde trafficking pathway

Identification of the site of action of RidL and the position of the LCV in the retrograde trafficking route are pivotal for an understanding of effector function (Figure 1). Further insights into the role of RidL were obtained by studying the effects of *ridL* deletion on the levels of retrograde trafficking components on LCVs (Finsel et al., 2013). Deletion of *ridL* does not affect the localization of the retromer subunits to the LCV, but the retrograde cargos Vps10 (a *D. discoideum* homolog of mammalian sortilin) and CI-MPR decorate a larger portion of LCVs. Moreover, the late endosomal/lysosomal marker LAMP1 increasingly localizes to LCVs in the absence of RidL. A larger portion of LCVs also stain positive for SNX1 and SNX2 in the absence of RidL, possibly because RidL competes with the SNXs for PtdIns(3)*P* binding. Additionally, the level of the retrograde trafficking regulator and Rab7 GAP TBC1D5 is increased on LCVs in absence of RidL (Bärlocher et al., 2017). Collectively, these findings suggest that RidL and its inhibition of retrograde trafficking results in decreased levels of retrograde cargos and some components of the retrograde machinery on LCVs.

Regarding the position of the LCV in the retrograde trafficking route different scenarios are conceivable. The LCV might represent a donor compartment in the retrograde trafficking route. Blockage of the pathway at the LCV by RidL would prevent vesicles from budding, elongation or scission, resulting in increased levels of retrograde components on the LCV in the presence of RidL (Figure 1). However, this is the opposite of what was observed, and therefore, it is unlikely that the LCV is a donor compartment in the retrograde pathway.

Alternatively, the LCV might be an acceptor compartment of retrograde trafficking vesicles (Figure 1). Blockage of incoming vesicles by RidL would prevent tethering, un-coating, attachment or fusion, leading to decreased levels of retrograde cargo, as observed (Finsel et al., 2013). Under these circumstances, the components of the retrograde transport machinery on the LCV would likely not be directly affected, unless incomplete vesicle un-coating or “kiss-and-run” interactions take place (Trahey and Hay, 2010). Furthermore, the presence of Rab4 (EE) and Rab11 (RE) on the LCV is also in agreement with the pathogen vacuole being a compartment accepting incoming retrograde trafficking from endosomes.

Interestingly, the retrograde cargos STxB and CTxB do not accumulate on LCVs, regardless of whether RidL is present or not (Finsel et al., 2013), and thus, the pathogen vacuole seems to serve as an acceptor compartment in some retrograde routes, e.g., the CI-MPR trafficking pathway, but not in others. At this point, we cannot rule out that the LCV is an acceptor compartment in the secretory pathway. Inhibition of retrograde trafficking by RidL might also reduce anterograde delivery of cargos such as MPR or Vps10/sortilin to the LCV by affecting the whole transport cycle (Riederer et al., 1994; Arighi et al., 2004). However, given the accumulation of retrograde machinery on LCVs such an indirect scenario seems less plausible.

## Conclusions and perspectives

The retrograde vesicle trafficking pathway appears to represent a pivotal component of cell-autonomous immunity, since several bacterial pathogens produce effector proteins subverting the pathway. *L. pneumophila* is unique in translocating an effector, RidL, which directly targets the eukaryotic retromer complex (Finsel et al., 2013). RidL binds the Vps29 retromer subunit, inhibits retrograde vesicle trafficking and promotes intracellular bacterial replication. The 29 kDa N-terminal domain of RidL adopts a novel fold including a hydrophobic β-hairpin, which binds Vps29, thereby displacing the Rab7 GAP TBC1D5 and contributing to subversion of retrograde trafficking in *L. pneumophila*-infected cells (Bärlocher et al., 2017).

The exact mode of action of RidL remains to be determined. While its 29 kDa N-terminal fragment is sufficient to displace TBC1D5, the C-terminal 102 kDa fragment of RidL likely harbors additional (perhaps catalytic) activities. Indeed, several *L. pneumophila* Icm/Dot substrates are large (>80 kDa) and represent multi-domain proteins with distinct (catalytic) activities (Itzen and Goody, 2011; Sherwood and Roy, 2013; Finsel and Hilbi, 2015). Thus, in addition to anchoring to Vps29 and displacing TBC1D5, RidL likely has other functions contributing to the inhibition of retrograde trafficking.

Moreover, the site of RidL action and the position of the LCV in the retrograde trafficking route are ill-defined at present. Cargos of the retrograde trafficking route (e.g., CI-MPR, sortilin) as well as the retromer subunits and the bacterial effector localize to LCVs. Yet, the retrograde toxin cargos STxB or CTxB cannot be detected on the LCV membrane, although their trafficking is affected by RidL. Due to its presence on the pathogen vacuole, RidL seems to act *in cis* (on the LCV) but might also function *in trans* (in a distance from the pathogen vacuole). Most available data is in agreement with the pathogen vacuole being an acceptor compartment in the retrograde trafficking pathway, such that RidL blocks the productive interaction of the LCV with incoming retrograde vesicles *in cis*, rather than inhibits the formation of retrograde transport vesicles at endosome exit sites *in trans* (Figure 1). Further studies will address the molecular function of RidL and the position of the LCV along the retrograde route.

## Author contributions

All authors listed have made a substantial, direct and intellectual contribution to the work, and approved it for publication.

### Conflict of interest statement

The authors declare that the research was conducted in the absence of any commercial or financial relationships that could be construed as a potential conflict of interest.

## Abbreviations

Icm/Dot: intracellular multiplication/defective organelle trafficking
LCV: *Legionella*-containing vacuole
OCRL: Oculocerebrorenal syndrome of Lowe
PI: phosphoinositide
PtdIns: phosphatidylinositol
SNARE: soluble *N*-ethylmaleimide-sensitive factor attachment receptor
SNX: sorting nexin
T4SS: type IV secretion system
TGN: *trans*-Golgi network.
